# Assessment and Correlation of the Vitamin D Level With Growth Status as Measured by the Cervical Vertebral Maturation Index and Insulin-Like Growth Factor 1 Level in 10–14-Year-Old Patients

**DOI:** 10.7759/cureus.109014

**Published:** 2026-05-17

**Authors:** Manika Jaiswal, Ananya Hazare, Himija Karia, Pritam R Khorgade, Nivedita Nandeshwar, Sanika M Thakur, Sangeeta Bhattacharya

**Affiliations:** 1 Department of Orthodontics and Dentofacial Orthopaedics, Ranjeet Deshmukh Dental College and Research Center, Nagpur, IND

**Keywords:** cervical vertebral maturation index, insulin-like growth factor 1, skeletal age, skeletal maturation, vitamin d level

## Abstract

Introduction

Growth assessment is a fundamental process in orthodontics as accurate determination of skeletal maturity guides the timing of treatment intervention. Biochemical and radiographic indicators can be used to evaluate growth status during adolescence. Insulin-like growth factor-1 (IGF-1) serves as a reliable biochemical marker of active growth, while the cervical vertebral maturation index (CVMI) provides a radiographic assessment of skeletal maturity. Vitamin D plays a vital role in bone metabolism and skeletal development. It may also influence growth mediators such as IGF-1. This study aims to assess and correlate levels of vitamin D with both IGF-1 and CVMI in 10-14-year-old patients.

Method

Patients who visited the Department of Orthodontics and Dentofacial Orthopaedics at Ranjeet Deshmukh Dental College and Research Centre of 10 to 14 years age were included in the study, provided they were not suffering from any systemic or endocrine disorders and had not consumed any vitamin D supplements. According to the established inclusion and exclusion criteria, a total of 50 subjects were selected for the study. Cervical vertebral maturation stages were assessed on a lateral cephalogram, which is routinely taken for orthodontic treatment. Two milliliters of blood samples were collected under all aseptic conditions with parental consent for assessment of IGF-1 by ELISA (DRG Diagnostics IGF-1 kit in Lisa Plus Elisa microplate reader) and vitamin D level by using ichroma vitamin D neo fluorescence Immunoassay.

Results

There was a statistically significant difference in vitamin D levels across CVMI stages (χ² = 14.813, p = 0.005). Mean vitamin D levels increased from Stage 2 (11.97 ± 0.96 ng/ml) to Stage 4 (24.54 ± 4.76 ng/ml), followed by a slight decline in Stage 5 (23.05 ± 4.88 ng/ml) and Stage 6 (19.10 ng/ml). Mean IGF-1 levels showed a consistent increasing trend: Stage 2 (19.57 ± 0.90 µg/dl), Stage 3 (24.77 ± 2.56 µg/dl), Stage 4 (28.78 ± 2.49 µg/dl), Stage 5 (29.25 ± 2.84 µg/dl), and Stage 6 (33.80 µg/dl). This demonstrates a progressive rise in IGF-1 levels with skeletal maturation. Considering the overall sample, there was a weak to moderately positive correlation between vitamin D and IGF-1 levels (ρ = 0.428).

Conclusion

Levels of vitamin D had significant variations among CVMI stages. IGF-1 showed a consistent increase with advancing stages of CVMI, reflecting its positive association with pubertal growth and skeletal maturation. Correlation analysis revealed a weak to moderately positive correlation between vitamin D and IGF-1 levels. Therefore, IGF-1 is a more reliable indicator of skeletal maturation as compared to vitamin D, while vitamin D may still play a supportive role in growth-related biologic processes and skeletal development.

## Introduction

Growth is one of the principal criteria to ground the treatment plan in orthodontics. For example, myofunctional and orthopedic treatment can be elicited only in periods of growth, and cessation of growth clearly indicates towards a surgical procedure for correction of skeletal deformity [1].

The cervical vertebral maturation index (CVMI) is a reliable indicator and a gold standard for individual skeletal maturity. It relies on the change in the morphological aspect, i.e., size and shape of the second, third, and fourth cervical vertebrae, indicating a specific stage of skeletal development with an arbitrary suggestion towards peak growth period [2].

Although cervical vertebral stages are considered a gold standard for skeletal age determination, there are limitations, such as subjective variations of clinicians in identifying the stages or the blend of one stage to another, which causes difficulty in the demarcation of borderline cases. Another limitation is frequent exposure of children to radiation for longitudinal radiographs through each stage of development [1].

To overcome the limitations of subjectivity and radiation exposure, biologic markers can be used for predicting growth as these are the components directly involved in bone metabolism and remodeling [3].

Insulin-like growth factor 1 (IGF-1) is produced in the liver and acts in synergy with the growth hormone, which is secreted by the pituitary gland. IGF-1 plays a crucial role in the growth of long bones and specifically growth at the mandibular condyle. Histological findings demonstrate that IGF-1 acts by accelerating the differentiation and synthesis of substrates required for the production of osteoblasts and chondroblasts [3].

Levels of IGF-1 are significantly higher in the pubertal stage as compared to the prepubertal and postpubertal stages; hence, IGF-1 can be a valuable tool for the skeletal maturity indicator [3].

Another modality that is often overlooked but is of prime importance for the growth and development of an individual is the nutritional status with adequate proportions of various minerals and vitamins. A vitamin that plays a critical role in bone metabolism and development of the skeleton is vitamin D, also known as an anti-rachitic hormone [4].

Deficiency of vitamin D is being recognized globally as an epidemic, even in high sunlight regions, contributing to a decreased absorption rate of the vitamin. The question is whether vitamin D deficiency could affect skeletal development, especially the jaw bones, and contribute to malocclusion [4].

A study performed by Leszczyszyn et al. assessed the impact of severity of vitamin D deficiency on the development of malocclusion. The results showed that 42.1% of deficient patients showed skeletal maldevelopment, and 46.5% had dentoalveolar malocclusion [5].

A study published by Carelli et al. estimated correlation of IGF-1 with skeletal maturity using hand wrist radiography and CVMI stages. The results of the study identified a moderately positive correlation of IGF-1 with skeletal maturity indicators [6].

Since published data indicate that the skeletal components are affected by the vitamin D level in the body, it becomes necessary to determine levels of vitamin D in growing individuals and then compare them with the gold standard of skeletal age estimation, along with the adjunctive means of biological markers of skeletal development.

## Materials and methods

This study aimed to assess the level of vitamin D in 50 subjects between 10 and 14 years and correlate it with skeletal maturation using the CVMI and IGF-1 level. Approval was obtained from the Institutional Ethics Committee (IEC/RDDC&RC/17/2024). The study was carried out from April 2024 to October 2025. Patients who visited the Department of Orthodontics and Dentofacial Orthopaedics for their orthodontic treatment were included in the study based on established inclusion and exclusion criteria.

Appropriate study armamentarium

Lateral cephalograms of subjects were evaluated for CVMI. Serum IGF-1 levels were estimated using enzyme-linked immunosorbent assay ELISA kits (DRG Products, Marburg, Germany) and a microplate reader (Lisa Plus, Aspen Diagnostics Pvt Ltd, New Delhi, India), and vitamin D levels were assessed using a fluorescence Immunoassay (FIA) analyzer (ichroma Vitamin D neo, Boditech Med Inc., Chuncheon-si, South Korea) with their corresponding test kits and micropipettes.

Inclusion and exclusion criteria

Patients who had taken lateral cephalograms for their orthodontic treatment, those who were willing to undergo this study, and those aged 10-14 years were included in the study. Patients with any systemic or endocrine disorders, craniofacial deformities, or who had consumed vitamin D supplements in the period of the last two years were excluded from the study.

Methods

Based on the inclusion and exclusion criteria, 50 subjects were chosen for the study who had lateral cephalograms taken for the treatment. With parental consent, 2ml of blood sample was withdrawn under all aseptic conditions.

Evaluation of Skeletal Development by Cervical Vertebral Maturation Stage

**Figure 1:**
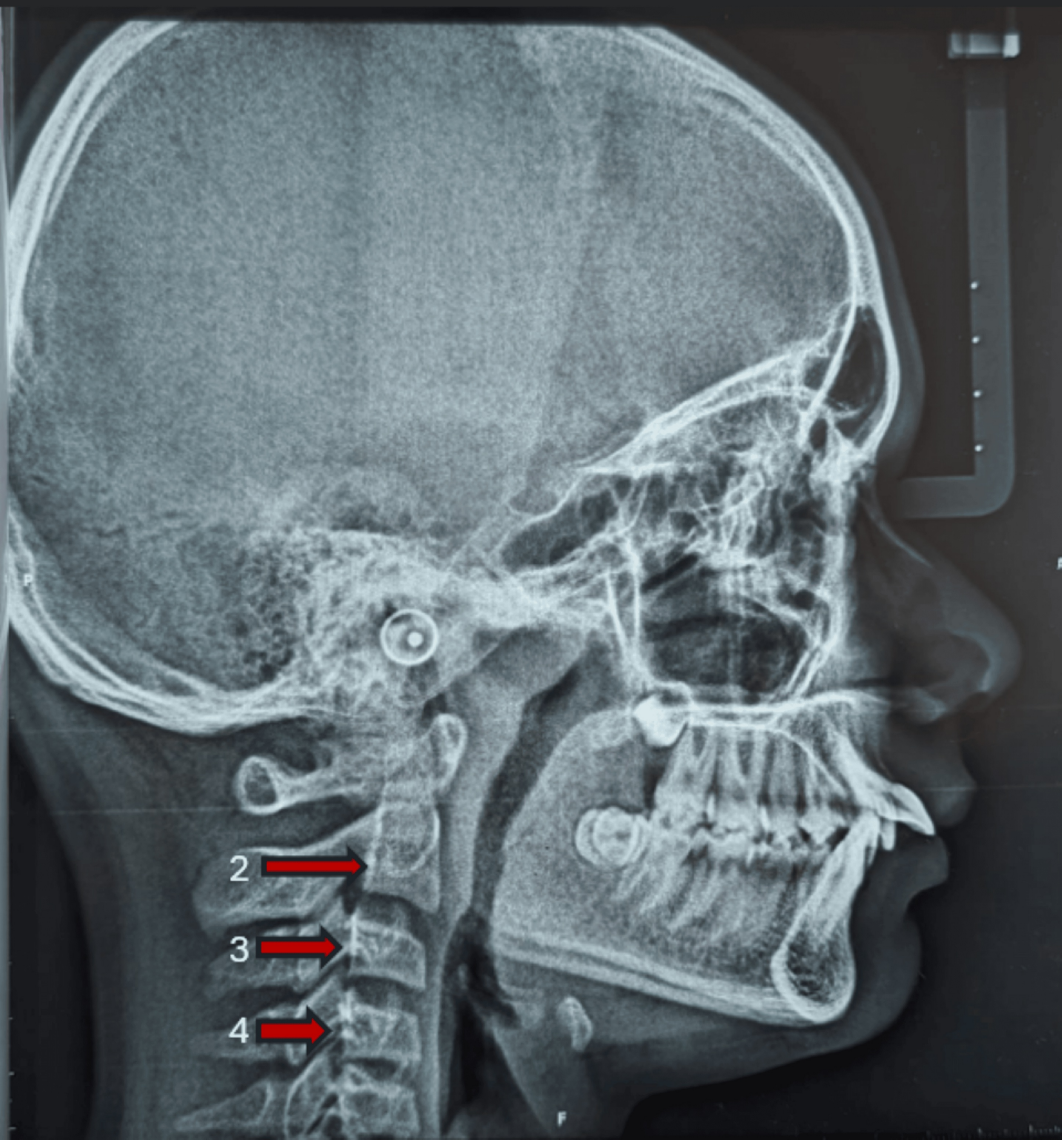
CV2, CV3 and CV4 CV: Cervical vertebra 2: Second cervical vertebra; 3: Third cervical vertebra; 4: Fourth cervical vertebra

This assessment involves evaluation of the morphological characteristics of the second, third, and fourth cervical vertebrae (CV2, CV3, and CV4) as visualized on lateral cephalometric radiographs (Figure 1). On the lateral cephalogram, the cervical vertebral maturation stage was assessed using CV2, CV3, and CV4 by evaluating (1) the presence of concavity on their inferior borders and (2) the shape of the vertebral bodies, progressing from trapezoidal to rectangular horizontal, square, and rectangular vertical forms. Based on these features, the patient was assigned to one of the six stages of cervical vertebral maturation (CVM1- CVM6) (Figure 1)

Evaluation of Insulin-Like Growth Factor-1

**Figure 2:**
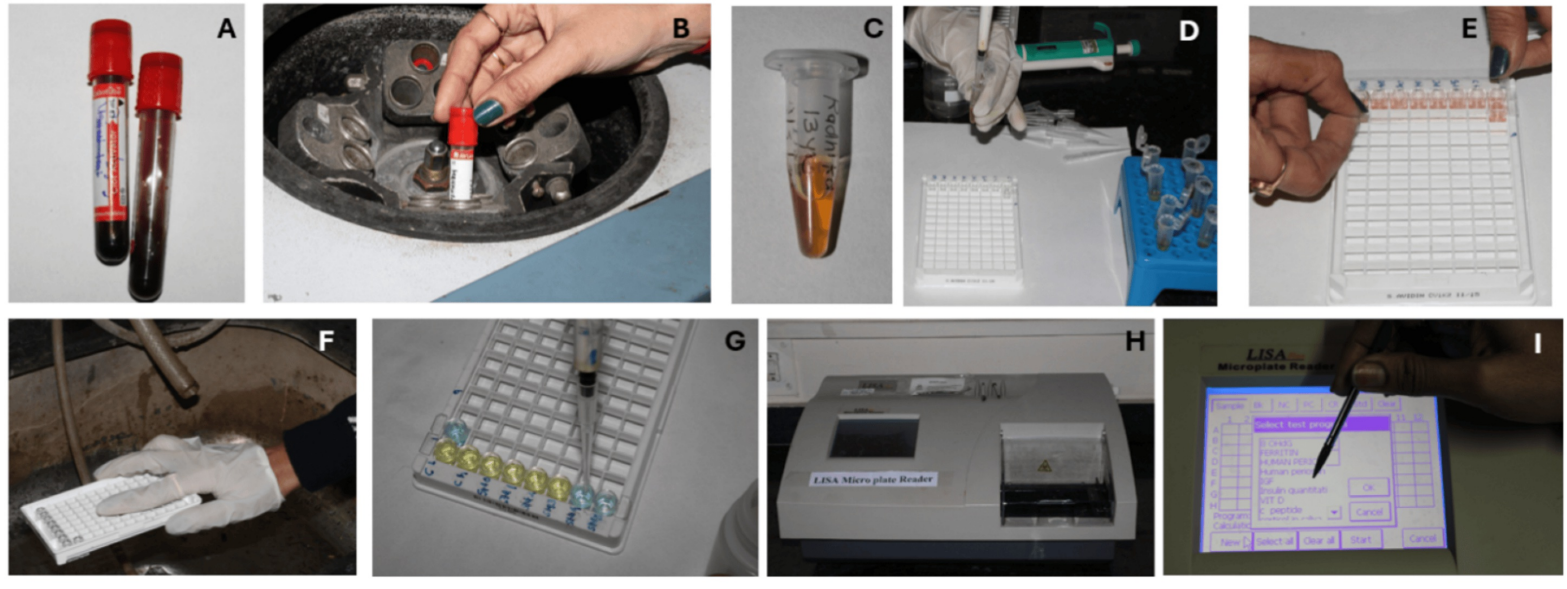
Estimation of IGF-1 through ELISA A. Sample collection; B. Centrifugation of the samples; C. Serum obtained; D. Plate loading; E. Incubation; F. Washing; G. Chromogenic substrate addition; H. ELISA microplate reader; I. IGF test on the ELISA reader IGF-1: Insulin-like growth factor 1

Blood samples were collected under aseptic conditions, followed by centrifugation to separate the serum (Figure 2). Standards, controls, and prepared serum samples were then added into microplate wells pre-coated with specific antibodies against IGF-1 from the IGF-1 kit (DRG Diagnostics, Marburg, Germany). The plate was incubated to allow IGF-1 present in the samples to bind to the immobilized antibodies. After incubation, the wells were thoroughly washed to remove any unbound components. An enzyme-linked detection antibody was subsequently added and incubated, facilitating the formation of an antibody-antigen-antibody complex. A chromogenic substrate was then introduced, leading to the development of a color proportional to the concentration of IGF-1 in the samples. Finally, the reaction was stopped by adding a stop solution, and the absorbance was measured using an ELISA reader (Lisa Plus- ELISA Microplate Reader).

Assessment of the Vitamin D Level

**Figure 3:**
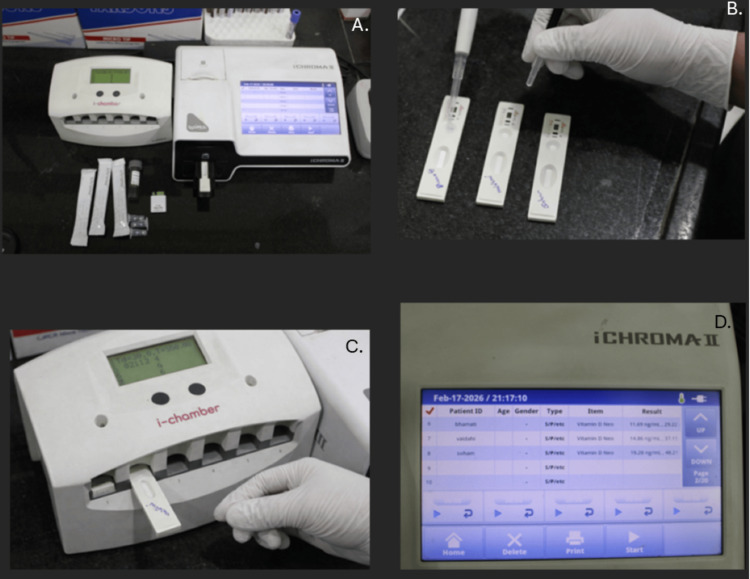
Assessment of the vitamin D level through FIA FIA: Fluorescence immunoassay A. Armamentarium; B. Loading cartridge with anti-vitamin D antibody and serum; C. Incubation; D. Reading through the FIA test

Serum vitamin D levels were evaluated using the FIA technique (Figure 3). Serum samples were added to anti-vitamin D antibodies, followed by the addition of a fluorescent reagent. The mixture was then incubated to allow the antigen-antibody reaction to occur. After incubation, the cartridge was inserted into the FIA analyzer (ichroma Vitamin D neo), which measured the fluorescence intensity and displayed the corresponding vitamin D concentration in ng/mL.

## Results

The purpose of this study is to determine if a correlation is present between CVMI, vitamin D level and IGF-1 level, contributing as indicators of skeletal growth.

The study was performed in 50 subjects with a chronological age of 10-14 years. The mean age increased progressively with advancing CVMI stages. Participants in Stage 2 had a mean age of 11.00 ± 1.73 years, followed by Stage 3 (11.38 ± 1.02 years), Stage 4 (12.71 ± 0.85 years), and Stage 5 (14.00 ± 0.00 years). Stage 6 included only one participant aged 14 years. Table 1* *confirms a positive association between skeletal maturation (CVMI stages) and chronological age. 

**Table 1:** Age distribution CVMI: Cervical vertebral maturation index

CVMI Stages	N	N (Percentage%)	Mean	SD
Stage 2	3	6%	11.00	1.73
Stage 3	21	42%	11.38	1.02
Stage 4	17	34%	12.71	0.85
Stage 5	8	16%	14.00	0.00
Stage 6	1	2%	14.00	-

Table* 2* demarcates that the Shapiro-Wilk test revealed levels of vitamin D were not normally distributed in CVMI Stage 3 (p = 0.005) and Stage 5 (p = 0.047), while Stage 2 (p = 0.712) and Stage 4 (p = 0.996) followed a normal distribution. In contrast, IGF-1 levels demonstrated normal distribution across all CVMI stages (p > 0.05). Based on these findings, non-parametric tests were appropriately used for vitamin D, while IGF-1 data justified parametric analysis. 

**Table 2:** Normality distribution CVMI: Cervical vertebral maturation index; IGF-1: insulin-like growth factor 1

Parameter	CVMI Stages	Statistic	df	Sig.
Vitamin D	Stage 2	0.977	3	0.712
	Stage 3	0.852	21	0.005
	Stage 4	0.988	17	0.996
	Stage 5	0.82	8	0.047
IGF-1	Stage 2	0.996	3	0.878
	Stage 3	0.976	21	0.859
	Stage 4	0.978	17	0.932
	Stage 5	0.954	8	0.747

There was a statistically significant difference in vitamin D levels across CVMI stages (χ² = 14.813, p = 0.005), as determined by the Kruskal-Wallis test. Mean vitamin D levels increased from Stage 2 (11.97 ± 0.96 ng/ml) to Stage 4 (24.54 ± 4.76 ng/ml), followed by a slight decline in Stage 5 (23.05 ± 4.88 ng/ml) and Stage 6 (19.10 ng/ml) (Table *3*). 

**Table 3:** Comparison of vitamin D levels (ng/ml) among different CVMI stages Kruskal Wallis test; * indicates a significant difference at p≤0.05 CVMI: Cervical vertebral maturation index

CVMI Stages	Mean	SD	χ^2^ Value	p-Value
Stage 2	11.97	0.96	14.813	0.005*
Stage 3	19.40	6.25		
Stage 4	24.54	4.76		
Stage 5	23.05	4.88		
Stage 6	19.10	-		

Post hoc Bonferroni analysis showed that the only statistically significant difference was between Stage 2 and Stage 4 (mean difference = 12.57, p = 0.008) (Table 4). Although differences between Stage 2 and Stage 5 approached significance (p = 0.058), they did not meet the threshold. All other pairwise comparisons were non-significant (p > 0.05). This indicates that vitamin D levels notably increase from early to mid-pubertal stages, but differences among later stages are less pronounced.

**Table 4:** Pairwise comparison of vitamin D levels among different CVMI stages Post hoc Bonferroni test; * indicates a significant difference at p≤0.05 CVMI: Cervical vertebral maturation index

CVMI Stages	Mean Difference	p-Value
Stage 2 vs Stage 3	7.43	0.410
Stage 2 vs Stage 4	12.57	0.008*
Stage 2 vs Stage 5	11.08	0.058
Stage 2 vs Stage 6	7.13	1.000
Stage 3 vs Stage 4	5.14	0.108
Stage 3 vs Stage 5	3.65	1.000
Stage 3 vs Stage 6	-0.3	1.000
Stage 4 vs Stage 5	-1.49	1.000
Stage 4 vs Stage 6	-5.44	1.000
Stage 5 vs Stage 6	-3.95	1.000

A highly significant difference in IGF-1 levels was observed across CVMI stages (F = 15.866, p < 0.001) (Table 5*)*. Mean IGF-1 levels showed a consistent increasing trend: Stage 2 (19.57 ± 0.90 µg/dl), Stage 3 (24.77 ± 2.56 µg/dl), Stage 4 (28.78 ± 2.49 µg/dl), Stage 5 (29.25 ± 2.84 µg/dl), and Stage 6 (33.80 µg/dl). This demonstrates a progressive rise in IGF-1 levels with skeletal maturation. 

**Table 5:** Comparison of IGF-1 levels (ug/dl) among different CVMI stages One-way ANOVA; * indicates a significant difference at p≤0.05 CVMI: Cervical vertebral maturation index; IGF-1: insulin-like growth factor 1

CVMI Stages	Mean	SD	F-Value	p-Value
Stage 2	19.57	0.90	15.866	<0.001*
Stage 3	24.77	2.56		
Stage 4	28.78	2.49		
Stage 5	29.25	2.84		
Stage 6	33.80	-		

Post hoc Tukey analysis revealed significant differences primarily between early and advancing stages. Stage 2 differed significantly from all other stages (p ≤ 0.014), and Stage 3 also showed significant differences when compared with Stages 4, 5, and 6 (p ≤ 0.009). However, no significant differences were observed among later stages (Stages 4, 5, and 6). These findings suggest that IGF-1 levels increase sharply during early pubertal stages and plateau in later stages of skeletal maturation (Table 6).

**Table 6:** Pairwise comparison of IGF-1 levels among different CVMI stages Post hoc Tukey test; * indicates a significant difference at p≤0.05 CVMI: Cervical vertebral maturation index

CVMI Stages	Mean Difference	p-Value
Stage 2 vs Stage 3	5.20	0.014*
Stage 2 vs Stage 4	9.21	<0.001*
Stage 2 vs Stage 5	9.68	<0.001*
Stage 2 vs Stage 6	14.23	<0.001*
Stage 3 vs Stage 4	4.01	<0.001*
Stage 3 vs Stage 5	4.48	<0.001*
Stage 3 vs Stage 6	9.03	0.009*
Stage 4 vs Stage 5	0.47	0.992
Stage 4 vs Stage 6	5.02	0.318
Stage 5 vs Stage 6	4.55	0.447

Spearman rank correlation analysis was performed to evaluate the relationship between vitamin D and IGF-1 levels across different CVMI stages. At Stage 2, a perfectly negative correlation was observed (ρ = -1.000); however, no p-value was computed due to the extremely small sample size (n = 3), making this finding statistically unreliable and not interpretable. At Stage 3, a weak positive correlation was noted (ρ = 0.337), but it was not statistically significant (p = 0.135), indicating no meaningful association between vitamin D and IGF-1 at this stage. At Stage 4, a moderate positive correlation was observed (ρ = 0.478), which approached statistical significance (p = 0.052). This suggests a trend toward a positive association, although it did not reach the conventional threshold for significance. At Stage 5, a moderate negative correlation was found (ρ = -0.405), but this was not statistically significant (p = 0.320), indicating no consistent relationship at this stage. When considering the overall sample, there was a moderate positive correlation between vitamin D and IGF-1 levels (ρ = 0.428), which was statistically significant (p = 0.002) (Table 7).

**Table 7:** Correlation between vitamin D levels and IGF-1 levels Spearman rank correlation test; * indicates a significant difference at p≤0.05 CVMI: Cervical vertebral maturation index; IGF-1: insulin-like growth factor 1

CVMI Stages	Spearman Rho	p-Value
Stage 2	-1.000	-
Stage 3	0.337	0.135
Stage 4	0.478	0.052
Stage 5	-0.405	0.320
Overall	0.428	0.002*

## Discussion

Various methods are used to identify the time of growth, namely, the chronological age, dental development, height and weight measurement, sexual maturation characteristics, and skeletal age [7]. Among all the parameters that help in interpreting the growth status of an individual, skeletal maturation contributes significantly to selecting an appropriate orthodontic treatment procedure. The CVMI determines craniofacial maturation based on the morphology of three cervical vertebral bodies visible on lateral cephalograms [2]. Although radiographic methods are well established and routinely used to assess skeletal maturity, they carry the drawbacks of subjective perception and low reproducibility [8]. 

Veena et al. in 2021 assessed in their systematic review whether non-invasive biomarkers prove to generate as reliable findings for skeletal maturity as other radiographic methods [8]. It was concluded that the level of biomarkers increased during pubertal growth spurt and provides a quantitative way to determine skeletal maturity [8].

In a study by Azarbakhsh et al. in 2022, it was noted that there is a high prevalence of vitamin D deficiency. As vitamin D is important for bone growth in children, evaluating its level along with skeletal maturation can help in planning growth modification treatments more effectively [9].

In this study, we simultaneously correlated skeletal maturity as determined by a radiographic method (CVMI) along with a non-radiographic biomarker (IGF-1) and assessed whether vitamin D could also be attributed as a significant parameter during skeletal development and maturity.

The results of the present study indicate that the mean chronological age increased progressively with advancing CVMI stages from Stage 2 to Stage 5. However, there was only one participant of Stage 6 CVMI included in the study. This study is supportive in accordance with several other Indian studies that have positively correlated cervical vertebral maturation stages with correspondingly increasing ages of the subjects. One of the studies by Mohan et al. in 2020 shows a significantly positive correlation of skeletal age with chronological age among all the subjects of the study [10].

As we have compared the level of IGF-1 in different CVMI stages, there was a consistent increasing trend from Stage 2 to Stage 6, with Stage 2 (19.57 ± 0.90 µg/dl), Stage 3 (24.77 ± 2.56 µg/dl), Stage 4 (28.78 ± 2.49 µg/dl), Stage 5 (29.25 ± 2.84 µg/dl), and Stage 6 (33.80 µg/dl). This demonstrates a progressive rise in IGF-1 levels with skeletal maturation. The findings of this study align in part with those of Carelli’s study, where IGF-1 was correlated with two of the radiographic methods to determine skeletal age, and it showed a moderately positive correlation with both of them [6]. However, when we performed a pairwise comparison, Stage 2 and Stage 3 of CVMI showed a significant difference as compared to the later stages. The findings of this investigation also agree partly with a study conducted by Masoud et al. in 2008, which concludes a significantly positive and linear correlation of IGF-1 with Cervical Vertebral stages from pre-pubertal to late-pubertal stages [11].

The findings of vitamin D across CVMI stages were not normally distributed, with a varying distribution through Stage 2 to Stage 4 and a slight decline in Stage 5 and Stage 6. All other pairwise comparisons for the vitamin D level in all the stages were also insignificant. However, subjects with severely deficient vitamin D levels correlate positively with early stages of skeletal maturation, indicating poor skeletal development. This study expands the results obtained by Azarbakhsh et al. in 2022, which states that less advanced skeletal maturation was observed in severe vitamin D deficiency, but dental age is unaffected by vitamin D level [9].

When we compared vitamin D with the biomarker of skeletal growth, Spearman's rank correlation analysis indicated that the trend towards positive correlation did not reach the conventional threshold. Considering the overall sample, a weak to moderately positive correlation existed between vitamin D and IGF-1 level. Similar to our study, Li et al. concluded in their study a nonlinear relationship between 25-hydroxy cholecalciferol and IGF-1 levels [12]. However, this study completely contradicts the results obtained by Ameri et al. in 2013, which states that vitamin D increases circulating IGF-1 levels, signifying a close association of these two parameters with one another [13].

Therefore, skeletal maturity demonstrates a positive association with age, with IGF-1 levels showing a consistent increase across CVMI stages, indicating its reliability as a maturation marker. In contrast, vitamin D levels exhibit variability and weaker, inconsistent correlations.

There are certain limitations to this study. The sample size was relatively small (n = 50), with unequal distribution across CVMI stages, particularly Stage 2 and Stage 6, which had very few subjects, thereby limiting the statistical power and reliability of stage-wise comparisons. Moreover, the assessment of CVMI is subject to inter-observer variability and subjective interpretation, which may affect the accuracy. Serum vitamin D levels can be influenced by external factors such as sunlight exposure, dietary habits, and seasonal variations, which were not controlled in this study. Lastly, only a single time point measurement of biomarkers was taken, rather than longitudinal tracking across growth phases.

## Conclusions

The present study shows a positive relation between age and skeletal maturity. Levels of vitamin D showed significant variations among CVMI stages, indicating a rise in levels from pre to pubertal stages, followed by a reduction in the later stages. However, subjects with severely deficient vitamin D levels correlate positively with early stages of skeletal maturation, indicating poor skeletal development irrespective of the chronological age. IGF-1 showed a consistent increase with advancing CVMI stages, reflecting its positive association with pubertal growth and skeletal maturation. Correlation analysis revealed a weak to moderately positive correlation between vitamin D and IGF-1 levels. Stage-wise correlations were inconsistent and largely non-significant, likely due to smaller sample sizes within individual groups.

Therefore, IGF-1 is a more reliable indicator of skeletal maturation as compared to vitamin D, and vitamin D may still play a supportive role in growth-related biologic processes and skeletal development.

## References

[1] Hassel B, Farman AG (1995). Skeletal maturation evaluation using cervical vertebrae. Am J Orthod Dentofacial Orthop.

[2] McNamara JA Jr, Franchi L (2018). The cervical vertebral maturation method: a user's guide. Angle Orthod.

[3] Gupta S, Jain S, Gupta P, Deoskar A (2012). Determining skeletal maturation using insulin-like growth factor I (IGF-I) test. Prog Orthod.

[4] Carmeliet G, Dermauw V, Bouillon R (2015). Vitamin D signaling in calcium and bone homeostasis: a delicate balance. Best Pract Res Clin Endocrinol Metab.

[5] Leszczyszyn A, Hnitecka S, Dominiak M (2021). Could vitamin D3 deficiency influence malocclusion development?. Nutrients.

[6] Carelli J, Mattos C, Morais ND (2021). Correlation between insulin-like growth factor I and skeletal maturity indicators. Glob Pediatr Health.

[7] Gupta M, Divyashree R, Abhilash P, A Bijle MN, Murali K (2013). Correlation between chronological age, dental age and skeletal age among monozygoyic and dizygotic twins. J Int Oral Health.

[8] Gv V, Tripathi T (2021). Non-invasive methods for the assessment of biomarkers and their correlation with radiographic maturity indicators - a scoping review. Prog Orthod.

[9] Azarbakhsh G, Iranparvar P, Tehranchi A, Moshfeghi M (2022). Relationship of vitamin D deficiency with cervical vertebral maturation and dental age in adolescents: a cross-sectional study. Int J Dent.

[10] Mohan R, Jain RK, Balakrishnan N (2020). Correlation between chronological age and skeletal age using cvmi and modified MP3 methods. Bioinformation.

[11] Masoud M, Masoud I, Kent RL Jr, Gowharji N, Cohen LE (2008). Assessing skeletal maturity by using blood spot insulin-like growth factor I (IGF-I) testing. Am J Orthod Dentofacial Orthop.

[12] Li W, Yu T (2023). Relationship between 25-hydroxyvitamin D and IGF1: a cross-sectional study of the Third National Health and Nutrition Examination Survey participants. J Health Popul Nutr.

[13] Ameri P, Giusti A, Boschetti M, Murialdo G, Minuto F, Ferone D (2013). Interactions between vitamin D and IGF-I: from physiology to clinical practice. Clin Endocrinol (Oxf).

